# In Vitro Screening of Thai Medicinal Plants Identifies Antiviral Candidates Against an Avian Herpesvirus

**DOI:** 10.3390/ani16142241

**Published:** 2026-07-20

**Authors:** Nisachon Apinda, Chaiwat Arjin, Nattanita Luekamlang, Nonpawit Sirirungruangsarn, Phonlakit Saetiew, Anucha Muenthaisong, Pongpisid Koonyosying, Kanokwan Sangkakam, Usanisa Konkhon, Panuwat Yamsakul, Wasana Chaisri, Nattawooti Sthitmatee

**Affiliations:** 1 Laboratory of Veterinary Vaccine and Biological Products, Faculty of Veterinary Medicine, Chiang Mai University, Chiang Mai 50100, Thailand; anucha.m@cmu.ac.th (A.M.); pongpisid_koo@nation.ac.th (P.K.); kanokwansangkakam@gmail.com (K.S.); usanisa2547.oum@gmail.com (U.K.); nattawooti.s@cmu.ac.th (N.S.); 2 Faculty of Veterinary Medicine, Chiang Mai University, Chiang Mai 50100, Thailand; nattanital.2312@gmail.com (N.L.); nonpawit.siri@gmail.com (N.S.); phorlakit1@gmail.com (P.S.); panuwat.y@cmu.ac.th (P.Y.); wasana.ch@cmu.ac.th (W.C.); 3 Department of Animal and Aquatic Sciences, Faculty of Agriculture, Chiang Mai University, 239, Huaykaew Road, Suthep, Muang, Chiang Mai 50200, Thailand; chaiwat.arjin@cmu.ac.th; 4 Office of Research Administration, Chiang Mai University, Chiang Mai 50200, Thailand; 5 Faculty of Medicine, Nation University, Chiang Mai 50210, Thailand

**Keywords:** avian herpesvirus, herpesvirus of turkey, antiviral activity, medicinal plants, *Caesalpinia sappan*, *Persicaria odorata*

## Abstract

Viral infections remain a major threat to poultry health and production worldwide, highlighting the need for novel antiviral strategies. This study evaluated the in vitro antiviral and virucidal activities of six Thai medicinal plant extracts against herpesvirus of turkey (HVT), a non-oncogenic avian herpesvirus widely used as a live vaccine vector and employed here as a preliminary in vitro screening model. Cytotoxicity, total phenolic content (TPC), and antioxidant capacity were assessed using the MTT, Folin–Ciocalteu, DPPH, ABTS, and FRAP assays. Among the tested extracts, *Caesalpinia sappan* (CS) and *Persicaria odorata* (PO) exhibited the highest TPC and antioxidant activities and were selected for further antiviral evaluation. Both extracts significantly reduced HVT infectivity, infectious viral titers, and intracellular viral DNA levels in DF-1 cells (*p* < 0.01). CS produced lower infectious viral titers (1.995 × 10^4^ TCID_50_/mL) than PO (8.24 × 10^4^ TCID_50_/mL); however, both extracts significantly reduced viral titers compared with the untreated control (1.78 × 10^6^ TCID_50_/mL). In the virucidal assay, pre-incubation of HVT with CS and PO significantly reduced infectious virus titers to 4.64 × 10^4^ and 2.15 × 10^4^ TCID_50_/mL, respectively, compared with the untreated control (1.47 × 10^6^ TCID_50_/mL) (*p* < 0.01). These findings identify *C. sappan* and *P. odorata* as promising sources of plant-derived compounds for future investigation as antiviral agents against avian herpesviruses. However, additional studies using virulent MDV-1, independent biological replicates, and in vivo models are required before their potential application to Marek’s disease control can be established.

## Simple Summary

Marek’s disease is a serious viral disease of chickens that causes substantial economic losses to the poultry industry worldwide. Although vaccination effectively reduces disease severity, it does not completely prevent viral infection or transmission, highlighting the need for complementary strategies to improve disease control. In this study, we evaluated six Thai medicinal plants for their ability to inhibit infection by herpesvirus of turkey (HVT), a non-oncogenic avian herpesvirus used here as a preliminary in vitro model for anti-avian-herpesvirus screening. We also assessed their antioxidant properties because antioxidant compounds are often associated with antiviral activity. Among the plants tested, *Caesalpinia sappan* (sappan wood) and *Persicaria odorata* (Vietnamese coriander) exhibited the strongest antioxidant activities and the greatest antiviral effects in cultured cells. These extracts reduced HVT infectivity, viral yield, and viral DNA levels under in vitro conditions. Our findings suggest that these two Thai medicinal plants contain natural compounds with promising antiviral potential. Further studies are needed to identify the active compounds, clarify their mechanisms of action, and confirm their efficacy against virulent Marek’s disease virus in vivo. This study provides new insights into the potential application of plant-derived compounds as complementary antiviral agents for improving poultry health and supporting Marek’s disease control.

## 1. Introduction

Marek’s disease (MD), caused by Marek’s disease virus (MDV), is a highly contagious T-cell lymphoproliferative disease affecting multiple organs and tissues in chickens [1]. It remains a major threat to the global poultry industry, causing substantial economic losses worldwide [2]. Global economic losses associated with MD are estimated to exceed USD 2 billion annually due to carcass condemnation, reduced egg production, and vaccination costs. In Thailand, MD outbreaks in the southern region were estimated to cause economic losses of approximately USD 295,893 in 2019. Vaccination remains the primary strategy for preventing and controlling MD. However, disease outbreaks continue to occur in vaccinated flocks [3,4], while field strains of MDV continue to evolve toward greater virulence.

Current MD vaccines reduce disease severity but do not prevent viral infection or shedding because they fail to induce sterile immunity [5]. Consequently, vaccinated birds may continue to harbor and transmit the virus, facilitating the emergence and circulation of more virulent strains [1]. Therefore, complementary strategies capable of inhibiting viral infection or replication are needed to enhance existing vaccination programs.

In recent years, Thai medicinal plants have attracted increasing attention as potential sources of bioactive compounds for disease prevention and control in both livestock and poultry. Many medicinal plants possess antioxidant, anti-inflammatory, and antiviral properties, making them attractive candidates for antiviral drug discovery. For example, *Houttuynia cordata* exhibits antiviral activity against herpes simplex virus type 1 (HSV-1) [6], whereas *Caesalpinia sappan* and *Tiliacora triandra* inhibit porcine reproductive and respiratory syndrome virus (PRRSV) replication [7]. In addition, *C. sappan* possesses well-documented anti-inflammatory and circulation-promoting properties [8], while *Senna siamea* has demonstrated analgesic and anti-inflammatory activities [9]. Likewise, *Piper sarmentosum* has been reported to exhibit anti-inflammatory, antipyretic, and gastroprotective properties in experimental animal models [10]. Despite these promising biological activities, the antiviral potential of Thai medicinal plants against avian herpesviruses remains largely unexplored.

Because experiments involving virulent MDV require specialized biocontainment and handling, herpesvirus of turkey (HVT), a non-oncogenic avian herpesvirus widely used as a live vaccine vector, provides a safe and reproducible preliminary model for evaluating anti-avian herpesvirus activity under in vitro conditions. However, HVT does not reproduce the oncogenic, immunosuppressive, or pathogenic characteristics of virulent MDV and therefore cannot fully predict antiviral efficacy against Marek’s disease virus. To our knowledge, no previous study has systematically evaluated the antiviral activities of these Thai medicinal plants against HVT as a preliminary avian herpesvirus screening model.

Therefore, this study aimed to evaluate the in vitro antiviral activity, virucidal activity, antioxidant capacity, and phytochemical profiles of six medicinal plants commonly found in northern Thailand, namely *Caesalpinia sappan*, *Senna siamea*, *Houttuynia cordata*, *Persicaria odorata*, *Piper sarmentosum*, and *Tiliacora triandra*. The findings of this study may facilitate the identification of plant-derived compounds with anti-avian-herpesvirus activity and provide a foundation for future investigations using virulent MDV and in vivo models to support the development of complementary antiviral strategies for poultry health.

## 2. Materials and Methods

### 2.1. Plant Materials and Extraction

Six Thai medicinal plants, *Caesalpinia sappan*, *Persicaria odorata*, *Tiliacora triandra*, *Senna siamea*, *Houttuynia cordata*, and *Piper sarmentosum*, were collected from Lamphun Province, Thailand. Fresh plant materials were cut into small pieces and dried in a hot-air oven at 60 °C for 48 h or until a constant weight was achieved. The dried materials were ground into a fine powder.

For extraction, 100 g of each powdered sample was extracted with 500 mL of 95% ethanol for 72 h with intermittent shaking every 12 h. The extracts were filtered through Whatman No. 1 filter paper (Cytiva, Marlborough, MA, USA), and the filtrates were concentrated under reduced pressure using a rotary evaporator. The crude extracts were stored at −20 °C until use.

### 2.2. Determination of Total Phenolic Content and Antioxidant Activity

Total phenolic content (TPC) was determined using the Folin–Ciocalteu method as previously described [11] and expressed as gallic acid equivalents (GAE).

Antioxidant activity was evaluated using the 2,2-diphenyl-1-picrylhydrazyl (DPPH) radical-scavenging assay [12], the ABTS radical cation decolorization assay [13], and the ferric reducing antioxidant power (FRAP) assay [14]. Antioxidant activities in the DPPH and ABTS assays were expressed as half-maximal inhibitory concentrations (IC_50_), whereas FRAP values were expressed as Fe^2+^ equivalents.

### 2.3. Cell Culture and Virus Propagation

DF-1 chicken embryo fibroblast cells were maintained in Dulbecco’s Modified Eagle Medium (DMEM; Gibco, Grand Island, NY, USA) supplemented with 10% fetal bovine serum (FBS; Gibco), 100 U/mL penicillin, and 100 µg/mL streptomycin. Cells were incubated at 37 °C in a humidified atmosphere containing 5% CO_2_.

The herpesvirus of turkey (HVT) strain Fc126 was propagated in confluent DF-1 cell monolayers maintained in DMEM supplemented with 2% FBS. Following the development of cytopathic effects, infected cultures were harvested by three freeze–thaw cycles to release cell-associated virus. Virus suspensions were aliquoted and stored at −80 °C until use. Infectious virus titers were determined by the immunoperoxidase monolayer assay (IPMA) [15], and expressed as TCID_50_/mL using the Spearman–Kärber method. Before each experiment, a single virus aliquot was thawed and diluted in maintenance medium to obtain the standardized virus inoculum (1 × 10^4^ TCID_50_/mL) based on the previously determined virus titer.

### 2.4. Cytotoxic Assay

Cytotoxicity was evaluated using the 3-(4,5-dimethylthiazol-2-yl)-2,5-diphenyltetrazolium bromide (MTT) assay [16]. DF-1 cells were seeded at 1 × 10^4^ cells per well in 96-well plates and incubated at 37 °C in a humidified atmosphere containing 5% CO_2_ until approximately 80–90% confluence. Cells were then exposed to serial two-fold dilutions of each plant extract for 72 h, while untreated cells cultured in complete medium served as the negative control. Following incubation, the cells were washed with phosphate-buffered saline (PBS) and incubated with MTT solution (5 mg/mL; Thermo Fisher Scientific, Waltham, MA, USA) for 4 h. The resulting formazan crystals were dissolved in 100 µL dimethyl sulfoxide (DMSO), and absorbance was measured at 540 nm. Cell viability was expressed relative to the untreated control, and the 50% cytotoxic concentration (CC_50_) was estimated using a four-parameter logistic (4PL) nonlinear regression model.

### 2.5. Virucidal Assay

Virucidal activity was evaluated using extract concentrations corresponding to their respective CC_50_ values. *Caesalpinia sappan* (67.87 µg/mL) and *Persicaria odorata* (407.20 µg/mL) extracts were prepared in maintenance medium (DMEM supplemented with 2% FBS) containing 0.1% (*v*/*v*) DMSO. An HVT inoculum (1 × 10^4^ TCID_50_/mL) was mixed with each extract and incubated for 1 h at 37 °C in a humidified atmosphere containing 5% CO_2_. Virus incubated in maintenance medium containing 2% FBS and 0.1% DMSO without plant extract served as the virus control, whereas uninfected DF-1 cells cultured under identical conditions served as the negative control. Following incubation, the virus–extract mixtures were inoculated onto confluent DF-1 cell monolayers cultured in 96-well plates and incubated for 48 h at 37 °C in a humidified atmosphere containing 5% CO_2_. The 48 h endpoint was selected because HVT infection was readily detectable in DF-1 cells while the cell monolayer remained intact and suitable for virus titration. Cell lysates and culture supernatants were subsequently collected for virus titration.

### 2.6. Inhibition of Viral Replication Assay

To evaluate antiviral activity during viral replication, DF-1 cells were seeded at 1 × 10^4^ cells per well in 96-well plates and incubated at 37 °C in a humidified atmosphere containing 5% CO_2_ until approximately 80–90% confluence. Cells were infected with HVT (1 × 10^4^ TCID_50_/mL) and incubated for 2 h to allow viral adsorption. The inoculum was then removed and replaced with maintenance medium containing *C. sappan* (67.87 µg/mL) or *P. odorata* (407.20 µg/mL). The final concentration of DMSO in all treatments, including the virus control, was maintained at 0.1% (*v*/*v*). Wells containing maintenance medium supplemented with 2% FBS and 0.1% DMSO without plant extract served as the virus control. Cells were further incubated for 72 h at 37 °C in a humidified atmosphere containing 5% CO_2_, after which cell lysates and culture supernatants were collected for virus titration.

### 2.7. Virus Titration

Infectious virus titers were determined using the immunoperoxidase monolayer assay (IPMA). Briefly, infected DF-1 cells were fixed with chilled methanol–acetone (1:1, *v*/*v*) for 7 min at room temperature and washed three times with phosphate-buffered saline (PBS). The cells were incubated with chicken anti-HVT serum diluted 1:100 in PBS for 1 h at 37 °C in a humidified atmosphere containing 5% CO_2_. After three washes with PBS, the cells were incubated with horseradish peroxidase (HRP)-conjugated anti-chicken IgG secondary antibody (1:2000 dilution; Alpha Diagnostic International, San Antonio, TX, USA) for 1 h at 37 °C. Following three additional washes with PBS, 60 µL of 3,3′-diaminobenzidine (DAB) substrate solution containing hydrogen peroxide (SeraCare, Milford, MA, USA) was added to each well and incubated for 5 min at room temperature. The reaction was terminated by rinsing the plates with distilled water. Viral antigen-positive cells were examined under a light microscope, and infectious virus titers were calculated as TCID_50_/mL using the Spearman–Kärber method.

### 2.8. Quantification of HVT DNA by qPCR

Intracellular HVT DNA levels were quantified by quantitative PCR (qPCR). DF-1 cells were infected with HVT and treated with *Caesalpinia sappan* or *Persicaria odorata* extracts at low (1/8 CC_50_) and high (1/4 CC_50_) concentrations. After 72 h of incubation, total DNA was extracted using the DNeasy Blood & Tissue Kit (Qiagen, Hilden, Germany) according to the manufacturer’s instructions.

Quantitative PCR was performed using SYBR Green chemistry (Meridian Bioscience, Cincinnati, OH, USA) with previously described primers targeting the HVT SORF1 gene [17]. The chicken ovalbumin (OVO) gene served as the endogenous reference gene for normalization [18]. Thermal cycling consisted of an initial denaturation at 95 °C for 2 min, followed by 40 cycles of denaturation at 95 °C for 5 s and annealing/extension at 60 °C for 30 s. Melt-curve analysis (65–95 °C) was performed to verify amplification specificity. Each sample was analyzed in technical triplicate, and quantification cycle (Cq) values were used for statistical analysis.

### 2.9. Statistical Analysis

Data are presented as mean ± SD. Differences among treatment groups were analyzed using one-way analysis of variance (ANOVA) followed by Tukey’s multiple-comparison test. Homogeneity of variance was assessed using the Brown–Forsythe test prior to one-way ANOVA. The 50% cytotoxic concentration (CC_50_) was estimated using nonlinear regression based on a four-parameter logistic (4PL) model. Statistical significance was defined as *p* < 0.05. All statistical analyses were performed using GraphPad Prism version 10.2.3 (GraphPad Software, San Diego, CA, USA).

## 3. Results

### 3.1. Cytotoxic Activities of Plant Extracts

**Figure 1:**
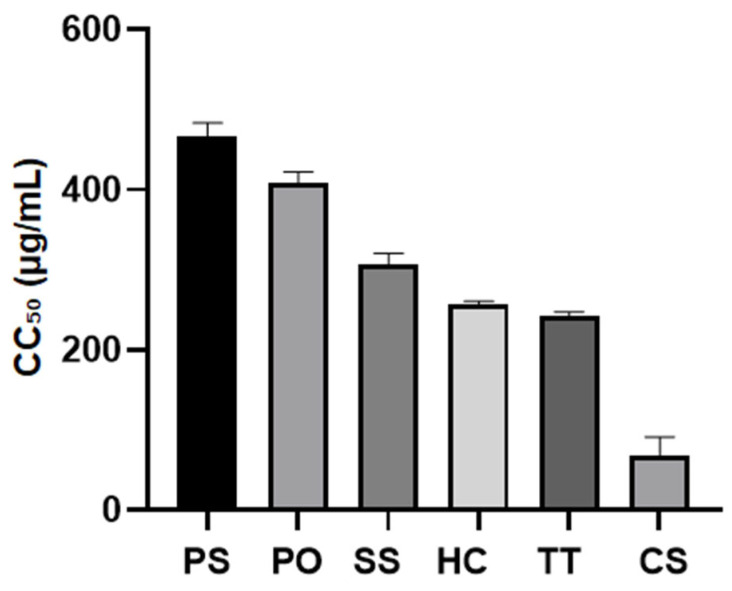
Cytotoxicity of six Thai medicinal plant extracts against DF-1 cells determined by the MTT assay. DF-1 cells were exposed to serial two-fold dilutions of each extract for 72 h. Cell viability was determined using the MTT assay, and CC_50_ values were estimated by nonlinear regression. Data are presented as mean ± SD. PS, *Piper sarmentosum*; PO, *Persicaria odorata*; SS, *Senna siamea*; HC, *Houttuynia cordata*; TT, *Tiliacora triandra*; CS, *Caesalpinia sappan*.

The cytotoxicity of six Thai medicinal plant extracts was evaluated in DF-1 cells using the MTT assay, and the 50% cytotoxic concentration (CC_50_) was determined (Figure 1). The extracts exhibited a wide range of cytotoxicity. Among the tested extracts, *Caesalpinia sappan* (CS) showed the lowest CC_50_ value (67.87 ± 23.01 µg/mL), indicating the highest cytotoxicity. *Tiliacora triandra* (TT), *Houttuynia cordata* (HC), and *Senna siamea* (SS) exhibited intermediate cytotoxicity, with CC_50_ values of 242.50 ± 4.64, 255.70 ± 4.74, and 305.60 ± 14.41 µg/mL, respectively. In contrast, *Persicaria odorata* (PO) and *Piper sarmentosum* (PS) exhibited the highest CC_50_ values, 407.20 ± 14.41 and 466.70 ± 16.59 µg/mL, respectively, indicating lower cytotoxicity than the other extracts. These findings demonstrate differences in cytotoxicity among the tested extracts, with *C. sappan* exhibiting the lowest CC_50_ value and *P. sarmentosum* the highest.

### 3.2. Phytochemical Contents of Thai Medicinal Plant Extracts

The total phenolic content (TPC) of the six plant extracts was determined using the Folin–Ciocalteu assay and expressed as milligrams of gallic acid equivalents per gram of extract (mg GAE/g) (Table 1). Considerable variation in TPC was observed among the extracts. *Caesalpinia sappan* contained the highest phenolic content (619.70 ± 63.88 mg GAE/g), followed by *Persicaria odorata* (410.12 ± 5.48 mg GAE/g) and *Piper sarmentosum* (179.45 ± 5.80 mg GAE/g). Lower TPC values were observed in *Tiliacora triandra* (97.60 ± 1.12 mg GAE/g), *Houttuynia cordata* (67.93 ± 2.49 mg GAE/g), and *Senna siamea* (57.15 ± 3.75 mg GAE/g). Among the six extracts, *C. sappan* contained the highest total phenolic content.

### 3.3. Antioxidant Activity

The antioxidant activities of the six Thai medicinal plant extracts were evaluated using the DPPH, ABTS, and FRAP assays (Table 1). In the DPPH assay, *C. sappan* exhibited the strongest radical-scavenging activity with the lowest IC_50_ value (16.95 ± 1.69 µg/mL), followed by *P. odorata* (20.07 ± 1.27 µg/mL). Moderate antioxidant activities were observed for *S. siamea* (251.10 ± 1.98 µg/mL) and *T. triandra* (332.53 ± 2.45 µg/mL), whereas *P. sarmentosum* (794.00 ± 2.08 µg/mL) and *H. cordata* (2060.00 ± 0.95 µg/mL) exhibited higher IC_50_ values, indicating lower radical-scavenging activity.

A similar trend was observed in the ABTS assay, where *C. sappan* exhibited the lowest IC_50_ value (420.56 ± 1.29 µg/mL), followed by *P. odorata* (585.24 ± 0.62 µg/mL). The remaining extracts showed substantially higher IC_50_ values. FRAP analysis also demonstrated the greatest reducing capacity in *C. sappan* (5.65 ± 0.11 mM Fe^2+^/g), followed by *P. odorata* (3.59 ± 0.05 mM Fe^2+^/g). Overall, antioxidant activity generally increased with total phenolic content among the tested extracts.

### 3.4. Virucidal Activity

**Figure 2:**
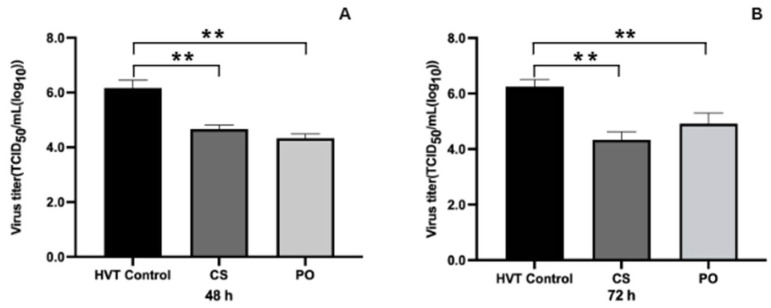
(**A**) Virucidal effects of *C. sappan* (CS) and *P. odorata* (PO) extracts against herpesvirus of turkey (HVT). Viral titers (log_10_ TCID_50_/mL) were determined after HVT was pre-incubated with *C. sappan* or *P. odorata* extracts at their respective CC_50_ concentrations for 1 h before infection of DF-1 cells. Both extracts significantly reduced viral titers compared with the untreated control (** *p* < 0.01). (**B**) Antiviral effects of *C. sappan* and *P. odorata* extracts on HVT replication in DF-1 cells. Viral titers (log_10_ TCID_50_/mL) were determined in HVT-infected DF-1 cells treated with CS or PO extracts at their respective CC_50_ concentrations 72 h post-infection. Both extracts significantly reduced viral titers compared with the untreated control (** *p* < 0.01).

**Figure 3:**
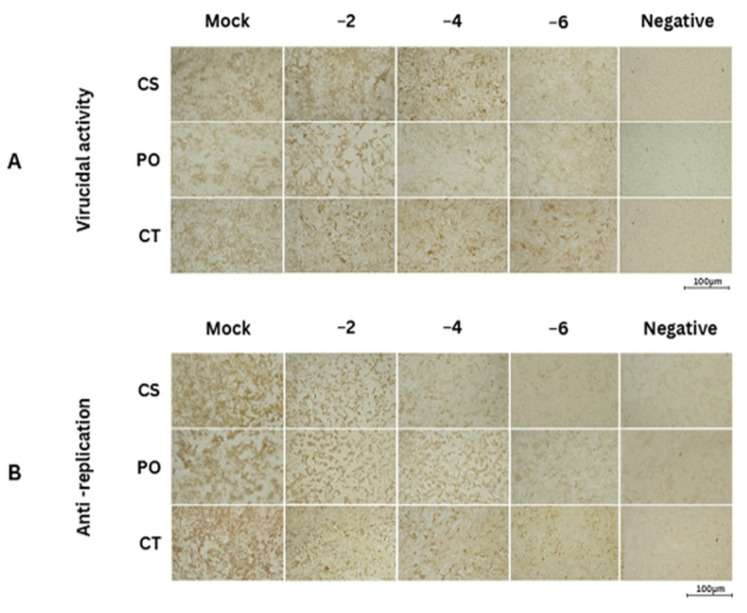
The IPMA images are representative qualitative observations provided to support the quantitative TCID_50_ results. (**A**) Virucidal effects of the extracts against HVT. IPMA images showing reduced viral antigen staining following virucidal treatment. The control group shows strong brown staining, while *C. sappan* (CS) and *P. odorata* (PO) treated samples exhibit markedly weaker staining. (**B**) Inhibitory effects of the extracts on HVT replication in DF-1 cells. IPMA images showing viral antigen distribution in HVT-infected DF-1 cells after treatment. Intense brown staining in the control group indicates active viral replication, whereas reduced staining in CS- and PO-treated cells demonstrates inhibition of viral antigen expression. Scale bar = 100 μm.

The virucidal activities of *C. sappan* and *P. odorata* were evaluated by incubating HVT with each extract before infection of DF-1 cells. Infectious virus titers were subsequently determined by IPMA and expressed as TCID_50_/mL (Figure 2A and Figure 3A).

Pre-incubation with *C. sappan* and *P. odorata* reduced infectious virus titers to 4.64 × 10^4^ and 2.15 × 10^4^ TCID_50_/mL, respectively, compared with 1.47 × 10^6^ TCID_50_/mL in the untreated control. Both extracts significantly reduced infectious virus titers compared with the untreated control (*p* < 0.01). No statistically significant difference was observed between the *C. sappan* and *P. odorata* treatment groups.

### 3.5. Antiviral Activity Against HVT Replication

The antiviral activities of *C. sappan* and *P. odorata* during HVT infection were evaluated by treating infected DF-1 cells with each extract after viral adsorption. Infectious virus titers were determined 72 h post-infection by IPMA and expressed as TCID_50_/mL (Figure 2B and Figure 3B).

Both extracts significantly reduced infectious virus titers compared with the untreated control (*p* < 0.01). Mean virus titers were 1.995 × 10^4^ TCID_50_/mL for *C. sappan*, 8.24 × 10^4^ TCID_50_/mL for *P. odorata*, and 1.78 × 10^6^ TCID_50_/mL for the untreated control. Although the mean infectious virus titer was lower in the *C. sappan* group than in the *P. odorata* group, the difference between the two extracts was not statistically significant.

### 3.6. Quantification of HVT DNA by qPCR

Intracellular HVT DNA levels were further evaluated by quantitative PCR targeting the viral SORF1 gene. The chicken OVO gene served as the endogenous reference and exhibited comparable Cq values among all treatment groups (25.25–25.88), indicating similar DNA input across samples.

**Figure 4:**
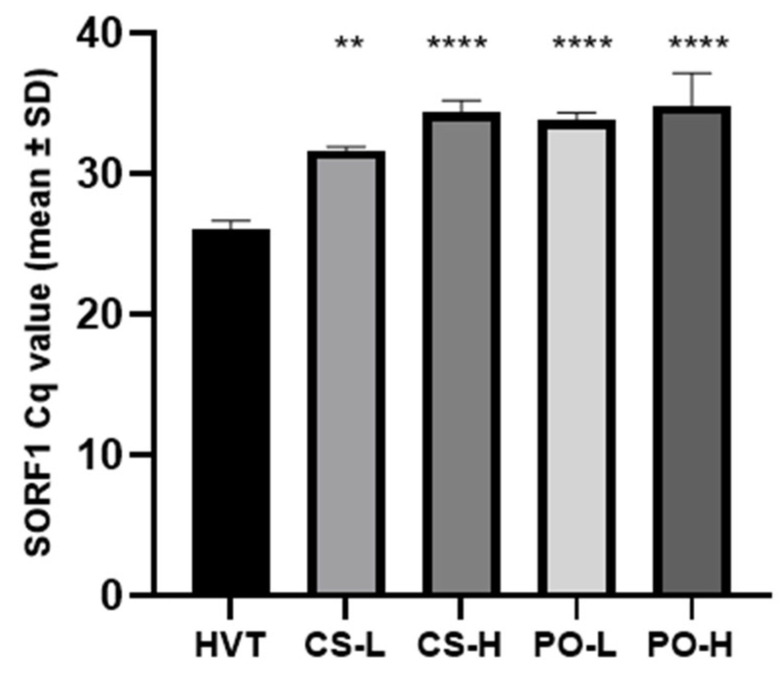
Effect of *C. sappan* (CS) and *P. odorata* (PO) extracts at low (L) and high (H) concentrations on HVT DNA levels measured by SORF1 qPCR. Data are presented as mean Cq ± SD of three technical replicates. Higher Cq values indicate lower HVT DNA levels. Statistical analysis was performed using one-way ANOVA followed by Tukey’s multiple comparison test. ** *p* < 0.01 and **** *p* < 0.0001 compared with the HVT control group.

Significant differences in SORF1 Cq values were observed among treatment groups (*p* < 0.0001). The infected HVT control exhibited the lowest mean Cq value (26.05 ± 0.59), corresponding to the highest intracellular HVT DNA level. In contrast, all extract-treated groups exhibited higher SORF1 Cq values, including CS-L (31.61 ± 0.32), CS-H (34.36 ± 0.86), PO-L (33.78 ± 0.57), and PO-H (34.84 ± 2.31), corresponding to lower intracellular HVT DNA levels than the infected control (Figure 4).

All extract-treated groups differed significantly from the infected control (*p* < 0.01). No significant differences were observed between CS-L and CS-H (*p* = 0.0946), PO-L and PO-H (*p* = 0.7985), or CS-H and PO-H (*p* = 0.9853). Overall, treatment with *C. sappan* or *P. odorata* was associated with significantly higher SORF1 Cq values than the infected control, consistent with lower intracellular HVT DNA levels.

## 4. Discussion

Among the six Thai medicinal plant extracts evaluated, *Caesalpinia sappan* and *Persicaria odorata* exhibited the highest antioxidant activities together with the greatest reduction in infectious virus titers and intracellular viral DNA levels in the HVT model. These findings demonstrate in vitro antiviral activity against HVT. However, the specific stages of the viral life cycle affected by these extracts remain unknown and require further investigation.

The reductions in infectious virus titers observed in the TCID_50_ assay were consistent with the increased SORF1 Cq values obtained by qPCR, suggesting reduced intracellular HVT DNA levels following treatment. Although higher extract concentrations generally produced higher Cq values, no statistically significant dose-dependent differences were observed. Additional studies using a broader concentration range and biological replicates are required to further characterize this relationship.

A positive association was observed between total phenolic content and antioxidant activity among the tested extracts. Similar associations have been reported previously for polyphenol-rich plant extracts with antiviral activity [19]. Although the present study did not identify the active antiviral compounds, the relatively high phenolic contents of *C. sappan* and *P. odorata* suggest that phenolic constituents may contribute to the observed activity. Identification of the responsible compounds will require phytochemical characterization and bioactivity-guided fractionation.

*C. sappan* has been widely investigated for its biological activities, including anti-inflammatory, antibacterial, and antiviral effects. Previous studies demonstrated that *C. sappan* extract inhibited porcine reproductive and respiratory syndrome virus (PRRSV) infection and replication, effects attributed to its high phenolic and flavonoid content [20]. Brazilin, catechin, and epicatechin have been identified as major constituents of the extract. Previous studies have suggested that brazilin may interfere with viral entry by interacting with host cell receptors, whereas catechin has been reported to interfere with viral glycoproteins involved in host-cell entry [21,22]. Although the antiviral mechanisms against avian herpesviruses remain unclear, these compounds may contribute to the antiviral activity observed in the present study.

*P. odorata* also demonstrated significant virucidal and antiviral effects against HVT. In addition to its traditional medicinal uses, previous studies have reported antibacterial, antifungal, anticancer, and antiviral activities associated with this herb [23]. *P. odorata* has been shown to inhibit chikungunya virus replication and contains several flavonoids and phenolic compounds with known antiviral properties [24]. Flavonoids such as quercetin, rutin, catechin, and kaempferol have been reported to inhibit a variety of DNA and RNA viruses through interference with viral entry, genome replication, and protein synthesis [25,26]. The antiviral activity observed in the present study is therefore consistent with previously reported biological properties of these compounds.

The virucidal assay showed that pre-incubation of HVT with either extract reduced infectious virus titers compared with the untreated control, indicating that antiviral activity occurred before infection of DF-1 cells. Together with the post-infection treatment results, these findings suggest that the extracts possess antiviral activity under both experimental conditions. However, the molecular mechanisms responsible for these effects remain to be determined.

The present study provides preliminary evidence that *Caesalpinia sappan* and *Persicaria odorata* possess in vitro antiviral activity against HVT, as demonstrated by reductions in infectious virus titers, viral antigen expression, and intracellular viral DNA levels. Because HVT was used as a preliminary avian herpesvirus screening model, these findings should be validated using virulent MDV-1 strains and independent biological replicates. Future studies should also identify the active antiviral constituents, elucidate their mechanisms of action, and evaluate their efficacy and safety in vivo. Collectively, these findings support further investigation of Thai medicinal plants as potential sources of antiviral compounds for avian herpesviruses.

## 5. Conclusions

Among the six Thai medicinal plant extracts evaluated, *Caesalpinia sappan* and *Persicaria odorata* exhibited the highest antioxidant activities together with significant virucidal and antiviral activities against herpesvirus of turkey (HVT), a non-oncogenic avian herpesvirus used as a preliminary in vitro screening model. Both extracts significantly reduced infectious virus titers, viral antigen expression, and intracellular viral DNA levels. These findings support further investigation of *C. sappan* and *P. odorata* as potential sources of plant-derived antiviral compounds against avian herpesviruses. Future studies should identify the active antiviral constituents, elucidate their mechanisms of action, and validate these findings using virulent MDV-1 strains and in vivo models.

## Figures and Tables

**Table 1:** Total phenolic content and antioxidant activities of six Thai medicinal plant extracts. Data are presented as mean ± SD (*n* = 3 independent measurements).

	Total Phenolic [mg GAE/g]	DPPH [IC_50_ µg/mL]	ABTS [IC_50_ µg/mL]	FRAP [mM Fe^2+^/g]
*Caesalpinia sappan*	619.70 ± 63.88	16.95 ± 1.69	420.56 ± 1.29	5.65 ± 0.11
*Persicaria odorata*	410.12 ± 5.48	20.07 ± 1.27	585.24 ± 0.62	3.59 ± 0.05
*Tiliacora triandra*	97.6 ± 1.12	332.53 ± 2.45	4019.98 ± 0.80	0.86 ± 0.01
*Senna siamea*	57.15 ± 3.75	251.1 ± 1.98	3820.08 ± 0.78	0.63 ± 0.003
*Houttuynia cordata*	67.93 ± 2.49	2060 ± 0.95	2329.32 ± 1.48	0.40 ± 0.02
*Piper sarmentosum*	179.45 ± 5.80	794 ± 2.08	2343.08 ± 0.36	1.01 ± 0.02

DPPH: 2,2-diphenyl-1-picrylhydrazyl, ABTS: 2,2-azino-bis (3-ethylbenzothiazo-line-6-sulfonic acid) diammonium salt, FRAP: ferric reducing antioxidant power, GAE: gallic acid equivalents, IC_50_: half maximal inhibitory concentration.

## Data Availability

The data presented in this study are available within the article. Additional data supporting the findings of this study are available from the corresponding author upon reasonable request.
